# Non-intubated deep paralysis: a new anaesthesia strategy for vocal cord polypectomy

**DOI:** 10.1186/s13741-023-00301-7

**Published:** 2023-04-19

**Authors:** Yichen Fan, Xiaoying Chi, Danyan Zhu, Jiemin Yin, Yaling Liu, Diansan Su

**Affiliations:** 1 Department of Anesthesiology, Renji Hospital Shanghai Jiaotong University School of Medicine, Shanghai, China; 2 Nursing department, Renji Hospital Shanghai Jiaotong University School of Medicine, Shanghai, China

**Keywords:** Non-intubated deep paralysis, Innovative anaesthesia strategy, Micro-laryngoscopic surgery, High-flow nasal oxygenation, Muscle relaxation

## Abstract

**Background:**

Vocal cord polyp is common otorhinolaryngological disease, traditionally treated by vocal cord polypectomy under a supporting laryngoscope with general anaesthesia. Although it is safe and controllable, it would cause some anaesthesia complications. Moreover, the complex process of general anaesthesia may significantly reduce surgical efficiency. Avoiding these problems remains an important issue.

**Methods:**

All patients were subjected to the standard non-intubated deep paralysis (NIDP) protocol consisting of four phases. An emergency plan was launched when NIDP cannot be implemented successfully. Patient characteristics, blood gas and monitoring data were collected during NIDP. Data concerning satisfaction, complications and duration of anaesthesia and recovery were collected to assess its effectiveness.

**Result:**

Among 20 enrolled patients, the success rate of NIDP was 95%. Only one patient failed in completing NIDP. Blood gas analysis revealed that the partial pressure of oxygen and carbon dioxide was maintained at safe levels. Monitoring during NIDP revealed fluctuations in mean arterial pressure between 110 and 70 mmHg, and the heart rate was stable at 60–100 beats per minute. The duration of anaesthesia and postoperative recovery were 13.0 ± 2.84 and 5.47 ± 1.97 min, respectively. All patients and surgeons were satisfied with NIDP, and no complications were detected before discharge.

**Conclusion:**

NIDP can be safely applied to patients and can replace general anaesthesia in vocal cord polypectomy. It can significantly reduce the duration of anaesthesia and postoperative recovery. No anaesthesia complications occurred without intubation, and patients and surgeons were satisfied with NIDP.

**Trial registrations:**

This single-centre, prospective study was registered on clinicaltrial.gov (NCT04247412) on 30^th^ July 2020.

## Introduction

Vocal cord polyps are exophytic benign lesions of the vocal cord and it is a common disease of otorhinolaryngology (Wang et al. 2013). Kim and his colleagues reported that the incidence of vocal cord polyps can reach as high as 2% in Korea, and the affected patients primarily comprised the age group of 30–60 years old (Kim et al. 2016). The major causes of this disease are excessive use of the vocal cord, upper respiratory inflammation, gastroesophageal reflux disease and smoking(Martins et al. 2011) (Byeon 2015). Voice therapy is a conservative treatment for vocal cord polyps, with success rates ranging from 30 to 40% (Lee et al. 2017). When conservative treatment cannot restore previous voice quality, vocal cord polypectomy is a common treatment used to remove vocal cord polyps (Rosen et al. 2009). The entire procedure of vocal cord polypectomy performed under general anaesthesia approximately consists of the following four steps: (1) anaesthesia with tracheal intubation, (2) placement of a supporting laryngoscope, (3) vocal cord polypectomy under the supporting laryngoscope, and (4) anaesthesia resuscitation. The key to successful surgery is to relax the pharyngeal muscles to completely expose the vocal cords with the supporting laryngoscope. The use of neuromuscular blocking agents is indispensable for surgery. Therefore, this type of micro-laryngoscopic surgery is generally performed under general anaesthesia with tracheal intubation (Morales-Angulo et al. 2000).

Although the application of general anaesthesia under tracheal intubation in vocal cord polypectomy is safe and controllable, it would still cause two problems clinically. First, the duration of anaesthesia is much longer than that of surgery, where it is over one hour from induction to awakening during the entire procedure of general anaesthesia, whereas it is only approximately several minutes in surgery. This tremendous difference significantly reduces surgical efficiency. Second, although tracheal intubation is essential in general anaesthesia, it could also raise a series of complications (e.g., blood pressure fluctuation, postoperative hoarseness, haemorrhage, and even negative-pressure pulmonary oedema) (Koide et al. 2020), which have a severe impact on the surgical process and the prognosis of patients.

A new anaesthesia strategy termed non-intubated deep paralysis (NIDP), which applies anaesthesia with deep neuromuscular blockade but without intubation, has been innovated to avoid these problems. The current study hypothesised that some specific types of surgery (e.g., vocal cord polypectomy), can be completed safely under this innovative anaesthesia strategy.

## Material and methods

This single-centre, prospective study was approved by the Ethics Committee of Renji Hospital (RA-2020–485) and registered on clinicaltrial.gov (NCT04247412).

### Study population

Patients who underwent elective vocal cord polypectomy were enrolled in this study. All patients provided written informed consent. The inclusion criteria were (1) patients aged 18–40 years old, (2) ability to communicate clearly, (3) estimated surgery time is less than 15 min, (4) patients undergoing elective unilateral vocal cord polypectomy, (5) willingness for study participation, and (6) ASA I–II grade. The exclusion criteria were (1) a history of neuromuscular disease, (2) body mass index > 30 kg·m^−2^, (3) severe gastrointestinal reflux disease, (4) pregnant or lactating women, (5) patients with a suspected difficult airway, and (6) allergy to specific agents used in NIDP protocol. All patients enrolled in this study signed the informed consent form before surgery.

### Study design

All patients were subjected to the standard NIDP protocol. In step 1, the pre-anesthetic phase, patients were enrolled according to the inclusion and exclusion criteria and instructed to fast for at least eight hours before surgery. No anti-acid agents were administered preoperatively accroding to the guidelines for preoperative fasting. After entering the operating room, pulse oxygen saturation (SpO_2_) and electrocardiography monitoring were routinely conducted. Arterial catheters were punctured to continuously monitor arterial blood pressure and blood gas. The patient had already undergone trans-nasal high-flow oxygenation (Airvo™ Fisher & Paykel Healthcare Corporation Limited, Auckland, New Zealand) for 5 min before anaesthesia induction, and the oxygen flow was set at 20 L·min^−1^. Video laryngoscope and tracheal tube were prepared for emergency conditions. Vasoactive agents were prepared before induction. Blood gas was checked before anaesthesia induction as the basic status of patients. Surgical instruments were also completely prepared in this phase. In step 2, the anaesthesia induction phase, propofol target controlled infusion (TCI) 5 µg·mL^−1^, remifentanil TCI 5 ng·mL^−1^, and midazolam 1 mg were administered for induction. When the patient lost consciousness, rocuronium 0.6 mg·kg^−1^ was administered, then the trans-nasal oxygen flow was increased and maintained to 60 L·min^−1^ until the patient was revived. In step 3, the anaesthesia maintenance phase, a supporting laryngoscope was placed by the surgeon to perform vocal cord polypectomy after 1 min of rocuronium administration. Blood gases were evaluated every 2 min during surgery to monitor the partial pressure of oxygen (PaO_2_) and partial pressure of carbon dioxide (PaCO_2_). Specific vasoactive agents (e.g., phenylephrine, urapidil, and esmolol) were used when the mean arterial pressure or heart rate were beyond the normal range of 70–110 mmHg and 60–100 beats per min. In step 4, the anaesthesia recovery phase, propofol TCI was stopped, and remifentanil, flumazenil 0.5 mg, and sugammadex sodium (BRIDION® MSD) 10 mg·kg^−1^ were administered after the completion of the surgery. When neuromuscular block was reversed and the patient recovered from unconsciousness, the trans-nasal oxygen flow was returned to 20 L·min^−1^. The patient continued to inhale high-flow oxygen for 5 min and was then transferred to post anesthesia care unit for further monitoring with mask oxygen inhalation. The final blood gas analysis was conducted 30 min postoperatively. The patient was sent back to the general ward if the Aldrete score was > 9.

**Fig. 1:**
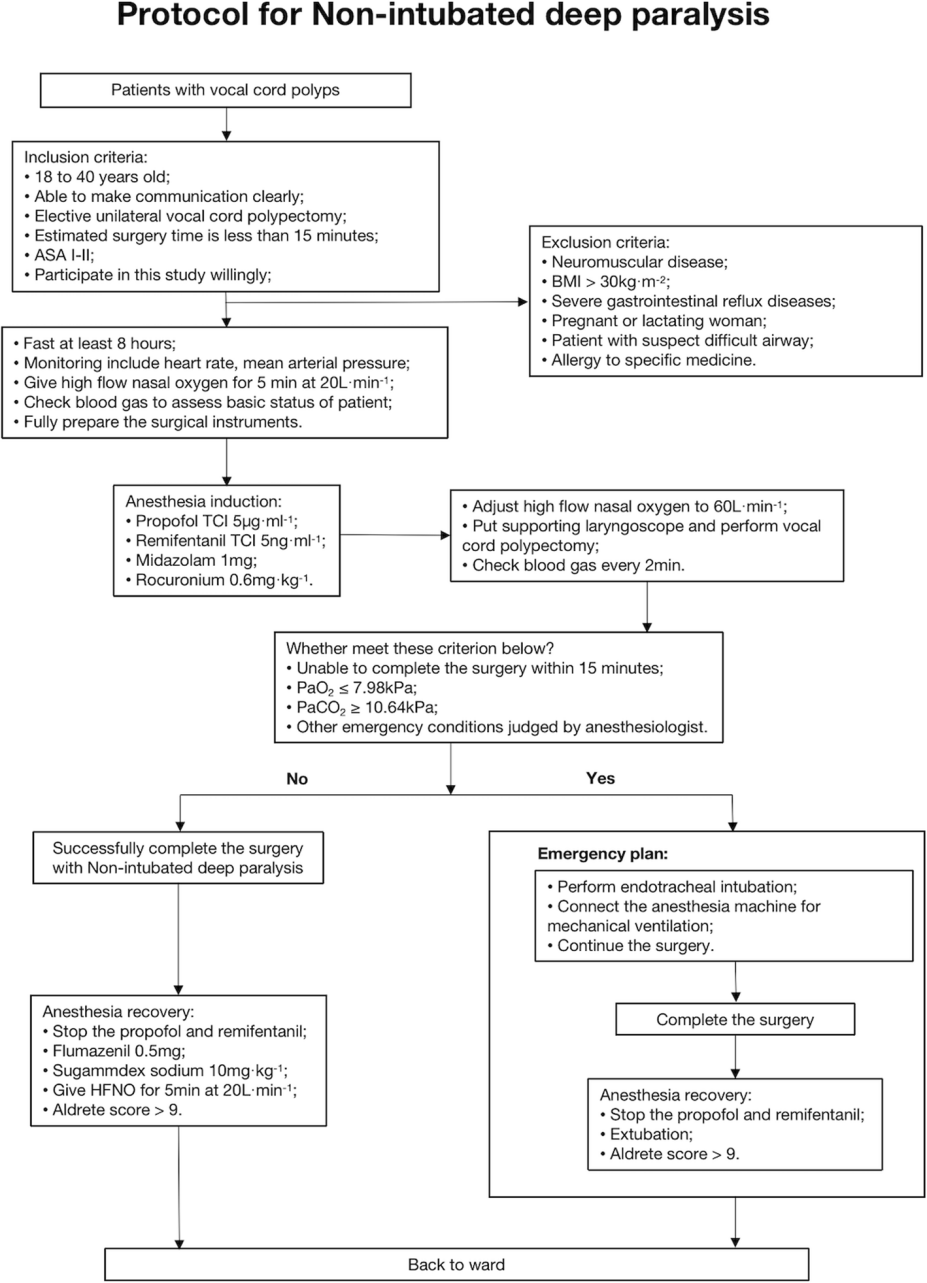
Protocol for Non-intubated deep paralysis. PaO_2_, arterial partial pressure of oxygen; PaCO_2_, arterial partial pressure of carbon dioxide; TCI, Target Controlled Infusion

Besides the standard protocol, an emergency plan is essential for NIDP. The criteria for implementing the emergency plan include (1) the surgery lasting for > 15 min, (2) hypoxemia (PaO_2_ ≤ 7.98 kPa), (3) hyper-capnia (PaCO_2_ ≥ 10.64 kPa), and (4) other emergency conditions judged by anaesthesiologist (e.g., severe intraoperative bleeding and unexpected gastro-esophageal reflux). The emergency plan should be implemented immediately to maintain the safety of the patient if these criteria are met. Implementing the emergency plan is relatively simple because all the anaesthetic agents have already been administered, and the anesthesiologist has to only insert the tracheal tube, perform NIDP, and then just convert it into general anaesthesia. Figure 1 shows the entire NIDP protocol.

### Primary endpoint

The primary endpoint in this study was the success rate of anaesthesia, which was defined as complete removal of polyps with no anaesthesia complication and no implementation of an emergency plan during the procedure.

### Secondary endpoints and safety measurements

The secondary endpoints included (1) postoperative hospitalization, (2) duration of anaesthesia, (3) duration of surgery, (4) duration of postoperative recovery, and (5) satisfaction of surgeons and patients. The duration of anaesthesia was defined as the time from the initiation of agent administration to patient recovery, and postoperative recovery duration was defined as the time from the end of surgery to patient recovery. Safety measurements included vital signs (e.g., mean arterial pressure, heart rate, blood gas, and post-anesthesia complications). Other perioperative variables included age, gender, and weight.

### Statistical analyses

The details of patient recruitment and exclusion were summarised. Patient characteristics (e.g., sex, age and basic disease) were recorded. Intraoperative and postoperative data were also collected.

SPSS 23.0 (IBM, Armonk, NY, USA) was used for statistical analyses. Categorical variables are expressed as numbers (n) or proportions (%), and numerical variables are expressed as mean ± standard deviation.

## Results

**Fig. 2:**
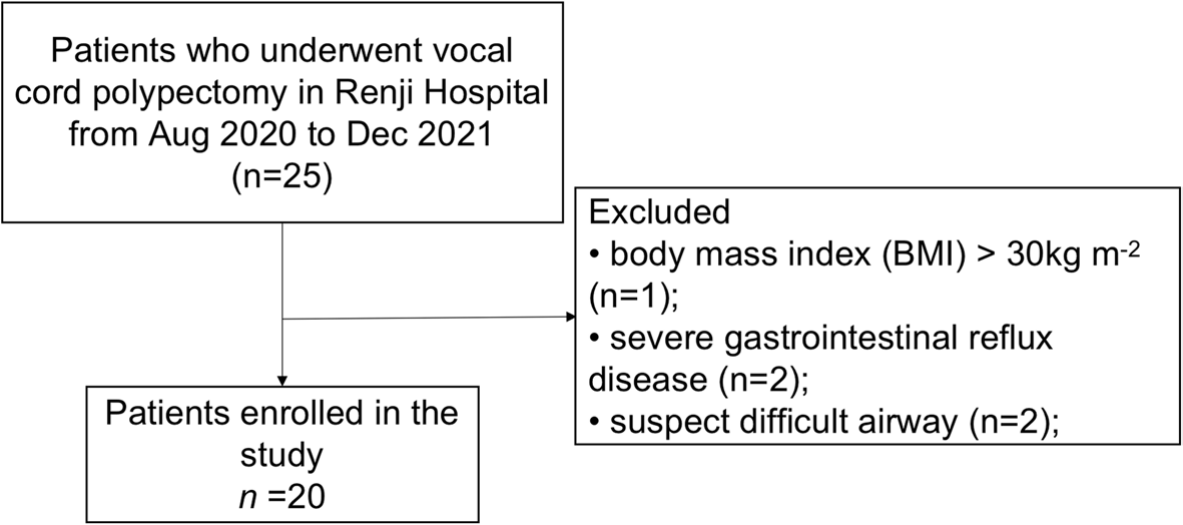
Flow diagram of study population

Of the 25 patients who underwent vocal cord polypectomy under a supporting laryngoscope between August 2020 and December 2021 at Renji Hospital, 5 were excluded based on the exclusion criteria and 20 patients were finally enrolled. The flow chart of patient recruitment and exclusion is depicted in Fig. 2.

Among the 20 study patients, NIDP was not successfully implemented in only one patient, indicating a 95% success rate of anaesthesia. That patient was female, 34 years old, weighing 70 kg and with a BMI of 26.3 kg·m^−2^. No abnormalities were found in the airway assessment. No comorbidity was noted before surgery, and the ASA classification was grade I. The anaesthesia induction phase was extremely stable. The primary cause of this failed case was an unexpected airway difficulty that caused failure to support laryngoscope placement, and the surgeon had attempted using various methods to insert the laryngoscope, but the vocal cords could not be still exposed. Blood gases were tested every 2 min. At 14 min of surgery, the PaCO_2_ and PaO_2_ levels were up to 11.3 and 20.22 kPa, respectively, with other indicators remaining in the normal range. As PaCO_2_ was extremely high and met the criterion of the emergency plan (PaCO_2_ ≥ 10.64 kPa), the NIDP protocol was suspended and the emergency plan was initiated. Tracheal intubation was performed following the emergency plan. After intubation, the tracheal tube was connected to the anaesthesia machine for mechanical ventilation, and the surgeon continued the operation. The entire surgery duration for this patient was 50 min, and the recovery duration was 30 min. Hence, the total anaesthesia duration for this patient was 80 min. No obvious anaesthesia complications, such as hoarseness, dislocation of cricoid cartilage and other vocal cord damage, occurred after surgery.

Characteristics and perioperative variables of patients are summarised in Table 1. The average age of patients was 34.7 ± 4.9 years old, the mean weight was 66.1 ± 8.5 kg and the average BMI was 23.2 ± 2.9 kg·m^−2^. All patients were classified as ASA grade I and diagnosed with unilateral vocal cord polyps. A history of hypertension was found in two patients. Regarding airway assessment, no patient had abnormalities, including mallampati class, mouth opening and thyromental distance.

**Table 1:** Characteristics and perioperative variables of patients.Values are mean (SD)

Variables	
Age (yr)	34.7(4.9)
Weight (kg)	66.1(8.5)
BMI (kg·m^−2^)	23.2(2.9)
Gender (Male / Female)	10 / 10
ASA (I / II)	20 / 0
Diagnosis	
Unilateral vocal cord polyp	20
Other Pharyngeal and laryngeal lesions	0
Co-morbidity	
Hypertension	2
Diabetes	0
OSAHS	0
Other disease	0
Mallampati class (I/II/III/IV)	12/8/0/0
Mouth opening 1/2/3^a^	0/0/20
Thyromental distance I/II/III^b^	19/1/0

*OSAHS* Obstructive sleep apnoea–hypopnea syndrome

^a^Mouth opening 1/2/3: 1, 1 finger; 2, 2 fingers; 3, 3 fingers;

^b^Thyromental distance I/II/III: I, > 6.5 cm; II, 6–6.5 cm; III, < 6 cm

**Fig. 3:**
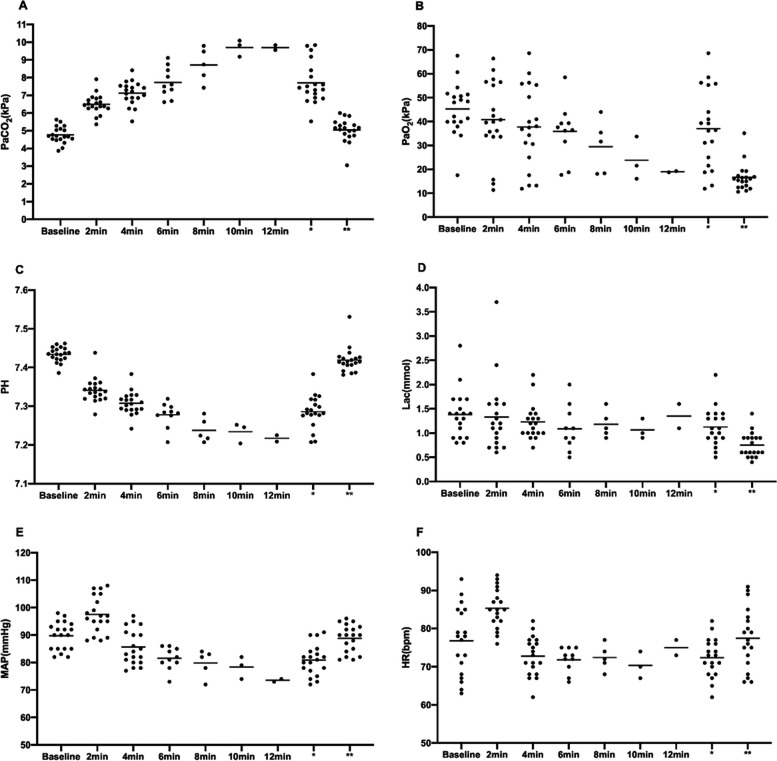
Blood gas result in PaCO_2_ (**A**), PaO_2_ (**B**), PH (**C**), Lac (**D**) and monitoring result in MAP (**E**), HR (**F**); The blood gas of all patients was maintained at a safe level during NIDP and mean arterial pressure fluctuated between 110 and 70 mmHg, and the heart rate was stable at 60–100 beats per min. Lac, lactic acid; MAP, mean arterial pressure; *end of surgery; **30 min postoperatively

The results of blood gas and monitoring data during NIDP, except for the failed case, are shown in Fig. 3. The PaO_2_ of all patients was maintained at a safe level during the entire anaesthesia process. The PaCO_2_ increased gradually during NIDP. The lactate and pH values were always in the safe range. Blood gas analysis, done 30 min postoperatively, revealed no abnormality in all patients. Mean arterial pressure and heart rate can be easily controlled by vasoactive agents at 110 to 70 mmHg and 60 to 100 beats per min, respectively.

The effectiveness evaluation data for successful NIDP are shown in Table 2. The duration of anaesthesia, surgery and postoperative recovery were 13.0 ± 2.84, 6.53 ± 2.2 and 5.47 ± 1.97 min, respectively.

**Table 2:** Effectiveness evaluation for Non-intubated deep paralysis. Values are mean (SD)

Success / Fail	19 / 1
Length of anesthesia (min)	13.0(2.84)
Length of surgery (min)	6.53(2.20)
Length of recovery (min)	5.47(1.97)
Postoperative hospitalization (h)	17.1(1.26)
Satisfaction of patients	
Yes	20
No	0
Satisfaction of surgeon	
Yes	20
No	0
Anesthesia complications	
Mild hoarseness	0
Dislocation of cricoid cartilage	0
Others	0

No anaesthesia-related complications were observed in these 20 patients. All patients and surgeons were satisfied with the NIDP protocol.

## Discussion

A new anaesthesia strategy, termed NIDP, a non-invasive anaesthesia technique, was innovated. A micro-laryngoscopic surgery (e.g., vocal cord polypectomy) can be performed safely under NIDP. Among the 20 enrolled patients, 19 completed NIDP, with only one case of failure. No anaesthesia complications were found in these 20 patients, and all patients and surgeons were satisfied with this anaesthesia strategy. All the safety parameters (e.g., mean arterial pressure, heart rate and blood gas) were in a relatively normal range.

General anaesthesia is the most reliable method of anaesthesia worldwide. It can be applied in various types of operations, including cardiovascular surgery, urological surgery, otorhinolaryngological procedures etc. Tracheal intubation under general anaesthesia, based on its advantage of being safe and controllable, has always been the primary anaesthesia method for vocal cord polypectomy. Nevertheless, due to the manipulation of tracheal intubation, the process of anaesthesia induction and recovery becomes relatively complex. Moreover, anaesthesia complications would sometimes occur. In contrast, the prominent advantage of NIDP is that it does not require tracheal intubation, avoiding the process of intubation, extubation and a series of complications. In addition, all anaesthetic agents used in NIDP are rapid-acting and metabolising or have certain antagonists. Therefore, the goal of rapid induction and prompt awakening can be achieved. However, NIDP may cause transient hypoxia and hyper-capnia, and hence its primary disadvantage is time limitation, implying that the anaesthesia duration cannot be maintained for too long time. Table 3 shows a comparison of general anaesthesia and NIDP.

**Table 3:** Comparison of general anesthesia and non-intubated deep paralysis

	General anesthesia	Non-intubated deep paralysis
Range of applications	Widely applied	Microlaryngoscopic surgery
Effect	Safe and controllable	Safe and controllable
Tracheal intubation	Yes	No
Anesthesia complications	Sometimes	Not found yet
Recovery time	Long	Short
Time-limition	No	Yes

NIDP implementation involves three principles. The first and most important principle is to avoid hypoxemia of patients during NIDP. To achieve this target, high-flow nasal oxygen (HFNO) was used to maintain the PaO_2_ of patients, which can provide high-flow humidification oxygen for patients with apnoea and prolong the safe apnoea time (Patel and Nouraei 2015). The mechanisms of HFNO include continuous positive airway pressure of the upper respiratory tract (Corley et al. 2011) and turbulence caused by high-flow oxygen on the supraglottis (Hermez et al. 2019). Previous studies also demonstrated that using HFNO can maintain oxygenation in patients with apnoea for 13–27 min (Huang et al. 2020). Furthermore, it is always important to address intubation issues in emergency cases.

The second principle of NIDP is the selection of anaesthetic agents that must be rapidly metabolised and antagonised. Propofol and remifentanil were used as sedative and analgesic agents during induction and maintenance in NIDP. These two drugs are rapidly metabolised, and their blood concentrations decreased rapidly after stopping the infusion. Rocha reported that the average recovery duration after combined use of propofol and remifentanil was only 8 min (Rocha et al. 2017). A supporting laryngoscope is essential for vocal cord polypectomy. Therefore, neuromuscular blocking agents are indispensable to relax pharyngeal muscles for supporting laryngoscope placement. Because of the fast onset and short action time, succinylcholine is an excellent neuromuscular blocking agent preferred by many anesthesiologists (Mostafa et al. 2022), but it has many adverse effects (e.g., myalgia, fasciculations, hyperkalemia and malignant hyperthermia) (Foldes et al. 1952), In this study, rocuronium, a rapid- and long-acting neuromuscular blocking agent, was administered to ensure a perfect neuromuscular blocking effect. Previous research has shown that the onset action time of rocuronium was approximately 60 s, but its average recovery duration was relatively long and can reach over 40 min (Khuenl-Brady and Sparr 1996). Fortunately, rocuronium has its specific antagonist, sugammadex sodium, a rapid-acting, effective and selective steroidal neuromuscular blocking antagonist that has already been approved for use by the US FDA in 2015. Sugammadex sodium is an improved γ-dextrin derivative that can reverse the effect of rocuronium by forming a compound with it (Takazawa et al. 2016). Its effectiveness has been widely recognised. Zwiers (Zwiers et al. 2011) found that the effect of sugammadex sodium linearly correlates with the dosage range of 0.1–16 mg·kg^−1^. Immediately after an intravenous injection of rocuronium 1.2 mg·kg^−1^, the administration of sugammadex sodium 16 mg·kg^−1^ can restore TOF ratio to 90% in 90 s. Extensive evidence shows that sugammadex sodium can rapidly and effectively reverse the deep neuromuscular blocking state of patients without obvious side effects (Varela and Lobato 2015). Abrishami also pointed out that the combined use of rocuronium and sugammadex can achieve the goal of rapid onset and recovery of neuromuscular blocking state (Abrishami et al. 2010). In the current study, sugammadex sodium 10 mg·kg^−1^ was used to antagonise the effect of rocuronium.

The third and final principle of NIDP is short surgery time. This is also an indication in NIDP. The duration of vocal cord polypectomy is very short, and the average duration was only 6.53 ± 2.2 min in this study. Patients cannot tolerate the condition of deep neuromuscular blocking without tracheal intubation for a long time. The limiting factor for NIDP may be not oxygenation but increasing in PaCO_2_ and decreasing in arterial PH (Fraioli et al. 1973). High PaCO_2_ can cause hyper-capnia that can produce a series of side effects (e.g., intracranial hypertension and immunosuppression), (Contreras et al. 2015) (Curley et al. 2010) It is vital to maintain PaCO_2_ at a safe level. High PaCO_2_ has been proved to increase cerebral blood flow and intracranial pressure rapidly, which would lead to headache, vomiting and even vision loss. Although Hickling reported that even a PaCO_2_ of 20 kPa was tolerable (Hickling and Permissive hypercapnia. 2002), this study set the limitation of PaCO_2_ at 10.64 kPa for the safty of patients. Moreover, Gustafsson reported that HFNO can also significantly delay the increase of PaCO_2_ (Gustafsson et al. 2017), and the removal rate of CO_2_ positively correlates with the velocity of high-flow oxygen (Möller et al. 1985). Hyperventilation are recommended preoperatively to reduce PaCO_2_. However, the operation duration must be as short as possible.

NIDP is an innovative anaesthesia strategy with important clinical values as follows: (1) without tracheal intubation, the risks and complications caused by general anaesthesia can be avoided considerably, and the comfort degree of patients can be increased; (2) combined with HFNO, it can maintain the oxygenation state of patients for a certain period without spontaneous breathing or mechanical ventilation, and NIDP may be safely applied in short-term surgeries as long as the protocol is followed; and (3) the use of specific anaesthetic agents and antagonists can achieve the aim of rapid induction and awakening. NIDP can shorten the duration of anaesthesia and completely improve the turnover efficiency of the operating room, and hence more patients can be treated. NIDP can be expanded to other short-term surgeries, including transurethral surgery, hysteroscopic surgery and haemorrhoid surgery in the future. Moreover, the inclusion criteria can be further optimised.

Due to the lack of a large sample size in this study, the application of NIDP is considered to have certain relative contraindications as follows: (1) the operation duration should not be too long (< 15 min) to avoid the obvious increase of PaCO_2_ or decrease of PaO_2_; (2) due to the use of HFNO, oxygen concentration in the respiratory tract is relatively high, and the use of an electrosurgical knife or laser should be avoided; (3) NIDP may be not suitable for patients with cardiopulmonary insufficiency; and (4) other contraindications include difficult airway, extreme obesity with a BMI of > 30 kg·m^−2^, gastrointestinal reflux and pregnant patients.

This study has two important limitations. First, it was a single-centre study with a small sample size. However, as this innovative anaesthesia strategy is still in the exploratory stage and to ensure patient safety, the sample size had to be controlled within a small range. A multi-centre study with a large sample size should be conducted to confirm the practicability of NIDP. An RCT should also be performed to verify the safety and effectiveness of NIDP compared with general anaesthesia. Second, the follow-up duration was quite short. Patients were followed up only until their discharge. Hence, this study lacked long-term statistics to evaluate the long-term impact of NIDP on patients.

## Conclusion

As an innovative anaesthesia technology, NIDP can be safely applied to patients and can replace general anaesthesia in some types of surgeries (e.g., vocal cord polypectomy). The major advantage of NIDP is its non-invasiveness. It can significantly reduce the duration of anaesthesia and postoperative recovery. Without intubation, no anaesthesia complications were noted, and patients and surgeons were satisfied with NIDP. However, the advantages of this anaesthesia strategy are still in the exploratory stage and require further verification.

## Data Availability

Datasets used or analysed during the current study are available from the corresponding author on reasonable request.
